# Intravenous Versus Oral Iron After Gastrointestinal Bleeding: A Systematic Review and Meta‐Analysis of Randomized Controlled Trials

**DOI:** 10.1002/jgh3.70225

**Published:** 2025-07-18

**Authors:** Mohamed Abuelazm, Ahmed Fares, Mohammad Adam, Yazan Sallam, Ahmed Mazen Amin, Hosam I. Taha, Mustafa Turkmani, Fouad Jaber

**Affiliations:** ^1^ Faculty of Medicine Tanta University Tanta Egypt; ^2^ Division of Gastroenterology and Hepatology Henry Ford Hospital Detroit Michigan USA; ^3^ Department of Internal Medicine University of Missouri‐Kansas City Kansas City USA; ^4^ Faculty of Medicine, Mansoura University Mansoura Egypt; ^5^ Faculty of Medicine Michigan State University East Lansing Michigan USA; ^6^ Division of Pulmonary and Critical Care University of Toledo Toledo Ohio USA; ^7^ Division of Gastroenterology and Hepatology Baylor College of Medicine Houston Texas USA

**Keywords:** anemia, hemorrhage, transfusion, variceal, varices

## Abstract

**Background & Objective:**

Few trials have compared the efficacy of intravenous (IV) iron repletion to oral repletion for patients with gastrointestinal bleeding (GIB). We aim to guide clinical decision‐making and optimize treatment strategies through the findings from these studies to provide a step closer to a consensus on the most effective approach to iron supplementation for patients with GIB.

**Methods:**

A systematic review and meta‐analysis synthesizing evidence from randomized controlled trials (RCTs) obtained from PubMed, Embase, CENTRAL, Scopus, and Web of Science from inception to April 2024. We used the fixed‐effects model to report dichotomous outcomes using risk ratio (RR) and continuous outcomes using mean difference (MD), with a 95% confidence interval (CI). PROSPERO ID: CRD42024542759.

**Results:**

Three RCTs that included 254 patients were included. IV iron was significantly associated with increased complete response (RR: 1.60 with 95% CI [1.24, 2.07], *p* < 0.01) compared to oral iron, with no significant difference between IV iron and oral iron in partial response (RR: 2.13 with 95% CI [0.60, 7.50], *p* = 0.24). IV iron was significantly associated with increased Hb concentration (MD: 1.45 g/dL with 95% CI [0.50, 2.40], *p* < 0.01) and ferritin change (MD: 220.02 μg/L with 95% CI [22.31, 417.73], *p* = 0.03) compared to oral iron. However, there was no significant difference between IV and oral iron in transferrin saturation (MD: 4.71% with 95% CI [−5.96, 15.38], *p* = 0.39).

**Conclusion:**

With uncertain evidence, IV iron demonstrated increased hemoglobin and ferritin concentrations and achieved complete response rates in patients with GIB.

## 1 Introduction

Gastrointestinal bleeding (GIB) is a medical emergency that requires prompt intervention. The incidence of upper GIB in the general population is estimated to range from 60 to 110 cases per 100 000 individuals annually, with hospital‐based data showing rates as high as 152 per 100 000 individuals [1]. Also, the incidence of lower GIB has been reported to be approximately 33–87 cases per 100 000 individuals per year [2]. Anemia is a common consequence of GIB, affecting up to 80% of patients, compared to a prevalence of 25% in the general population. Among these cases, iron deficiency anemia (IDA) is the most frequent, accounting for up to 60% of anemia in GIB [1, 3]. Additionally, acute blood loss anemia contributes significantly, as ongoing hemorrhage and inadequate iron replacement can lead to progressive iron depletion, even in the absence of overt IDA [4, 5].

Traditionally, oral iron has been favored for its accessibility and cost‐effectiveness and is considered first‐line for treating IDA. However, oral iron's gastrointestinal (GI) side effects, such as constipation and gastric irritation, often lead to poor compliance [6]. This is further confounded by lower bioavailability than intravenous (IV) formulations and is particularly important with GIB, given the pre‐existing abdominal symptoms. IV iron supplementation carries the benefit of fewer GI side effects and avoids poor absorption through bypassing the GI tract [7]. It also provides faster administration of large doses when rapid repletion is required, which is critical in GIB [8], providing a mode of therapy with greater compliance and completion [9]. However, IV iron is not without risks. Theoretically, IV iron might worsen cardiovascular outcomes due to its impact on oxidative stress and could potentially increase the risk of infections. Nevertheless, these concerns have not been supported by clinical trials [10].

Few studies have compared the effectiveness of IV iron repletion to oral repletion for patients with GIB [11, 12, 13]. Bager and Dahlerup [12] showed in their randomized controlled trial (RCT) that IV iron was more effective than oral iron for upper GIB in ensuring sufficient iron stores. Still, there was no significant difference in raising hemoglobin (Hb) concentration. Another RCT by Ferrer‐Barcelo et al. showed that IV ferric carboxymaltose (FCM) was superior to oral ferrous sulfate (FeSulf) in rapidly normalizing Hb concentration and improving iron status parameters among patients with anemia following acute GIB [13]. Finally, the RCT by Tabish et al. [11] also demonstrated that IV iron supplementation significantly outperformed oral iron in increasing Hb concentration, normalizing iron stores, and improving anemia in patients with cirrhosis after variceal bleeding.

This systematic review aims to guide clinical decision‐making and optimize treatment strategies through the findings from these studies to provide a step closer to a consensus on the most efficacious approach to iron supplementation for patients with GIB.

## 2 Methodology

### 2.1 Protocol Registration

This systematic review and meta‐analysis was completed using the Preferred Reporting Items for Systematic Reviews and Meta‐Analyses (PRISMA) statement [14] and the Cochrane Handbook for systematic reviews and meta‐analyses [15]. A prospectively registered protocol is available on PROSPERO with ID: CRD42024542759.

### 2.2 Data Sources & Search Strategy

We searched Embase, Scopus, PubMed, Web of Science, and CENTRAL databases from inception until April 2024. We performed a search for studies in English with the following keywords: (“intravenous iron” OR “ferric carboxymaltose” OR “IV iron” OR” iron derisomaltose” OR “iron supplement*” OR “iron therapy” OR “iron sucrose” OR “iron isomaltoside” OR “ferric gluconate”) AND (gastrointestinal* OR GI OR varices OR variceal OR non‐variceal) AND (bleed* OR hemorrhage OR hemorrhage). The detailed search strings for each database and results are outlined in (see Table S1).

### 2.3 Eligibility Criteria

We included RCTs that followed the following PICO criteria: population (P), adult patients with GIB, irrespective of the etiology, and confirmed anemia, with Hb < 12 g/dL; intervention (I), IV iron preparations, regardless of type and dose; control (C), oral iron preparations, regardless of type and dose; and outcomes: our primary outcome was complete response (defined as patients who reached Hb concentration ≥ 12 g/dL) and partial response (defined as patients with Hb increase ≥ 2 g/dL versus baseline). Also, our secondary outcomes included hemoglobin change, ferritin change, transferrin saturation (TSAT), and the incidence of hypophosphatemia, gastrointestinal adverse events, and rebleeding.

We excluded studies for the following reasons: (1) observational or descriptive studies, such as retrospective cohort or case–control studies; (2) crossover study design; (3) studies that included patients with hematological malignancies; (4) pregnancy; (5) patients with anemia of chronic disease, such as co‐existing kidney disease; (6) insufficient data for inclusion.

### 2.4 Study Selection

Using the Covidence online tool, two authors (XX and XX) reviewed the studies independently. They made an independent decision to include the studies in two steps: title/abstract screening and full‐text screening. The senior author (XX) reviewed the assessment and made the final decision.

### 2.5 Data Extraction

A pilot extraction was conducted after obtaining the full texts of the relevant publications to design an Excel (Microsoft, USA) extraction form. This was divided into three sections: summary characteristics of the included trials (first author name, year of publication, country, study design, number of centers, sample size, GIB type, intervention details, main inclusion criteria, follow‐up duration, and primary outcome); baseline characteristics of the included participants (number of patients in each group, age, gender, number of patients who received transfusion, baseline iron, baseline ferritin, and baseline TSAT); and the outcome data as previously described. Two authors (XX and XX) extracted data from the included studies using a standardized data extraction form. After initial extraction by both authors, the senior author (XX) reviewed and confirmed data validity. Any conflicts were discussed and settled.

### 2.6 Risk of Bias and Certainty of Evidence

The risk of bias in included studies was assessed using the revised Cochrane Collaboration tool for RCTs (ROB 2) [16]. Two reviewers evaluated each study independently for selection, performance, reporting, attrition, and overall biases, with disagreements resolved through consensus. To investigate the certainty of evidence, Grading of Recommendations Assessment, Development, and Evaluation (GRADE) recommendations [17, 18] were followed, considering inconsistency, imprecision, indirectness, publication bias, and risk of bias. The evaluation was carried out for each outcome, and the decisions were justified and documented. Any discrepancies were settled through discussion.

### 2.7 Statistical Analysis

We used R software version 4.3.1 for the statistical analysis. For dichotomous outcomes, the risk ratio (RR) was used. In contrast, for continuous outcomes, the mean difference (MD) was used, both with a 95% confidence interval (CI) using the random‐effects model when there was a significant heterogeneity (*I* ^2^ > 50%) and the common‐effect model when heterogeneity was not significant (*I* ^2^ < 50%). Heterogeneity was assessed using chi‐square and I‐square tests. The chi‐square test shows the presence of heterogeneity, and the I‐square test shows the degree of heterogeneity. We used an alpha level below 0.1 for the chi‐square test to interpret significant heterogeneity. Finally, we used the trial sequential analysis (TSA) [19] to detect the conclusiveness and reliability of the data from pooled studies and to determine if the current sample size is sufficient to reach a solid conclusion. We used an alpha error of 0.05, a beta error of 80% power, and an anticipated RR reduction of 30% in dichotomous outcomes.

### 2.8 Ethics Approval and Consent to Participate

Ethics approval and consent to participate were not applicable, as this study is a systematic review and meta‐analysis that did not involve direct participation of human subjects.

## 3 Results

### 3.1 Search Results and Study Selection

**FIGURE 1:**
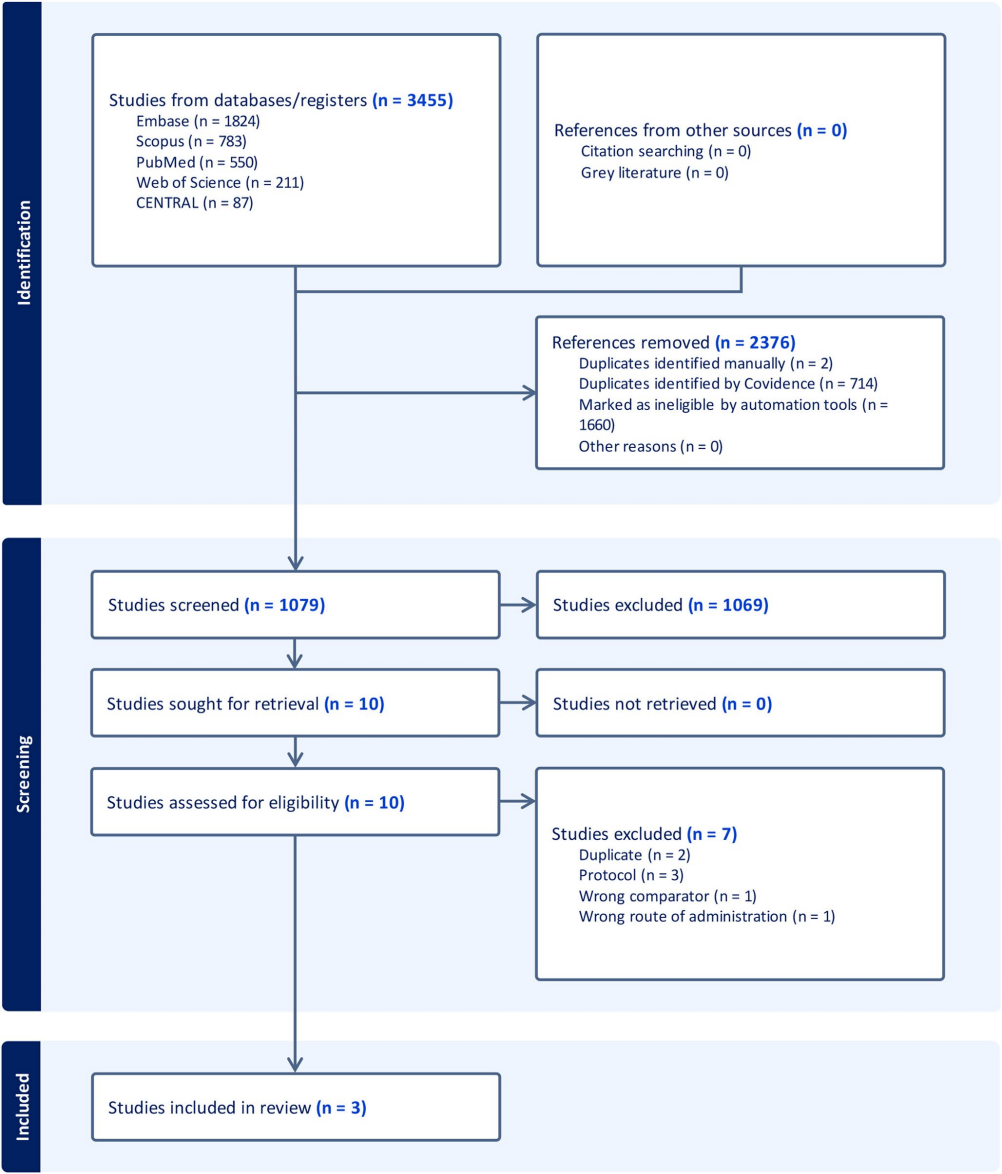
PRISMA flow chart of the screening process.

During the search process, 3455 studies were retrieved from databases and considered for screening based on their titles and abstracts. After filtering out duplicates and papers that did not match the inclusion criteria, 10 full‐text articles were evaluated. Seven of these were found to be irrelevant and eliminated, leaving three RCTs to include in the quantitative analysis (Figure 1).

### 3.2 Characteristics of Included Studies

Full‐text screening identified three RCTs, including 254 patients, two in Europe and one in India [11, 12, 13]. Bager and Dahlerup (Denmark) was a double‐blinded, single‐center trial using a block‐randomized design with dark, non‐transparent bags and black intravenous lines to maintain blinding [12]. Ferrer‐Barcelo et al. [13] (Spain) was an open‐label, single‐center study that assigned participants using an alternating sequence based on enrollment order. Tabish et al. [11] (India) was also an open‐label, single‐center trial, with randomization performed using a computer‐generated random number. Among the three studies, only Bager and Dahlerup [12] implemented double blinding. The overall proportion of female participants was 44% (73/163), and the average age ranged from 45 to 71. The intervention arm received two doses of FCM, except in Bager and Dahlerup [12] where only a single dose was administered, while control groups received daily oral iron. Additionally, Tabish et al. [11] focused on patients with variceal bleeding, whereas the other two studies examined non‐variceal bleeding [12, 13]. A summary of the included studies and participants' characteristics is shown in (Tables 1 and 2).

**TABLE 1:** Summary characteristics of the included participants.

Study, year	Bager and Dahlerup [12]	Ferrer‐Barceló et al. [13]	Tabish et al. [11]
Study design	Double blinded RCT	Open‐label RCT	Open‐label RCT
Study duration	13 weeks	6 weeks	12 weeks
Country, study center	Denmark/Aarhus University Hospital	Spain/University General Hospital of Valencia	India/All India Institute of Medical Sciences
Total no. of patients	97	65	92
Type of GIB	Non‐variceal acute upper GIB	Acute Non‐variceal GIB	Acute Variceal GIB
Main inclusion criteria	Age > 18 + Hb < 12 (Men) or < 13 (Women), at 48 h post endoscopy	Age > 18 + Hb < 10 (at discharge)	Liver cirrhosis + age ≥ 18 years + Hb < 10 g/dL + iron deficiency
Primary outcome	Hb difference	% of patients who reached Hb concentration ≥ 12 g/dL or ≥ 13 g/dL in women and men, respectively	Hb difference
Secondary outcome	(1) % of Hb increases greater than 2 g/dL. (2)% of patients who reached the mean Hb reference values (13.5 g/dL for women, 15.0 g/dL for men). % of restoration of iron stores.	(1) Percentage of patients with Hb increase ≥ 2 g/dL vs. baseline. (2) Iiron status normalization (% TSAT ≥ 25%). (3) S. Ferritin.	(1) % of patients with improvement in anemia (hemoglobin > 12 g/dL). (2) normalization of iron stores (Serum ferritin > 100 ng/mL). (3) increase in hemoglobin at 4 weeks.
Intervention	Single dose of Ferric carboxymaltose	Two doses of Ferric carboxymaltose	Two doses of Ferric carboxymaltose
Control	Ferrous sulphate 100 mg BID	Ferrous sulphate 325 mg BID	Folipic‐Z, carbonyl iron
Duration of therapy	13 weeks	6 weeks	12 weeks
Partial response definition	Hemoglobin increment ≥ 2 g/dL from baseline	Hemoglobin increment ≥ 2 g/dL from baseline	NM.
Complete response definition	(Hemoglobin ≥ 13.5 g/dL [women], ≥ 15 g/dL [men]) after 13 weeks	(Hemoglobin ≥ 12 g/dL [women], ≥ 13 g/dL [men]) after 6 weeks	Improvement in anemia (hemoglobin > 12 g/dL) after 12 weeks

Abbreviations: BID, twice per day; GIB, gastrointestinal bleeding; Hb, hemoglobin; NM, not mentioned; RCT, randomized controlled trial.

**TABLE 2:** Baseline characteristics of the participants.

Study ID	Bager and Dahlerup [12]		Ferrer‐Barceló et al. [13]		Tabish et al. [11]	
Arm	IV iron	Oral iron	IV iron	Oral iron	IV iron	Oral iron
Total no. of patients	42	41	29	32	48	44
Age (Mean, SD)	69 ± 14.25	71 ± 18	57.8 ± 15.3	62.5 ± 18.3	45.96 ± 12.07	46.11 ± 12.25
Male/Female (No.)	23/19	21/20	17/12	22/10	43/5	37/7
Number who received blood transfusions	33	32	16	18	NA	NA
Baseline iron (mcg/dL)	NA	NA	NA	NA	29	30
Baseline Ferritin (ng/mL)	161	174	85.4	78.5	30	40
Baseline Transferrin saturation (%)	20	21	16	14.9	7	9

Abbreviations: IV, intravenous; No., number; SD, standard deviation.

### 3.3 Risk of Bias and Certainty of Evidence

**FIGURE 2:**
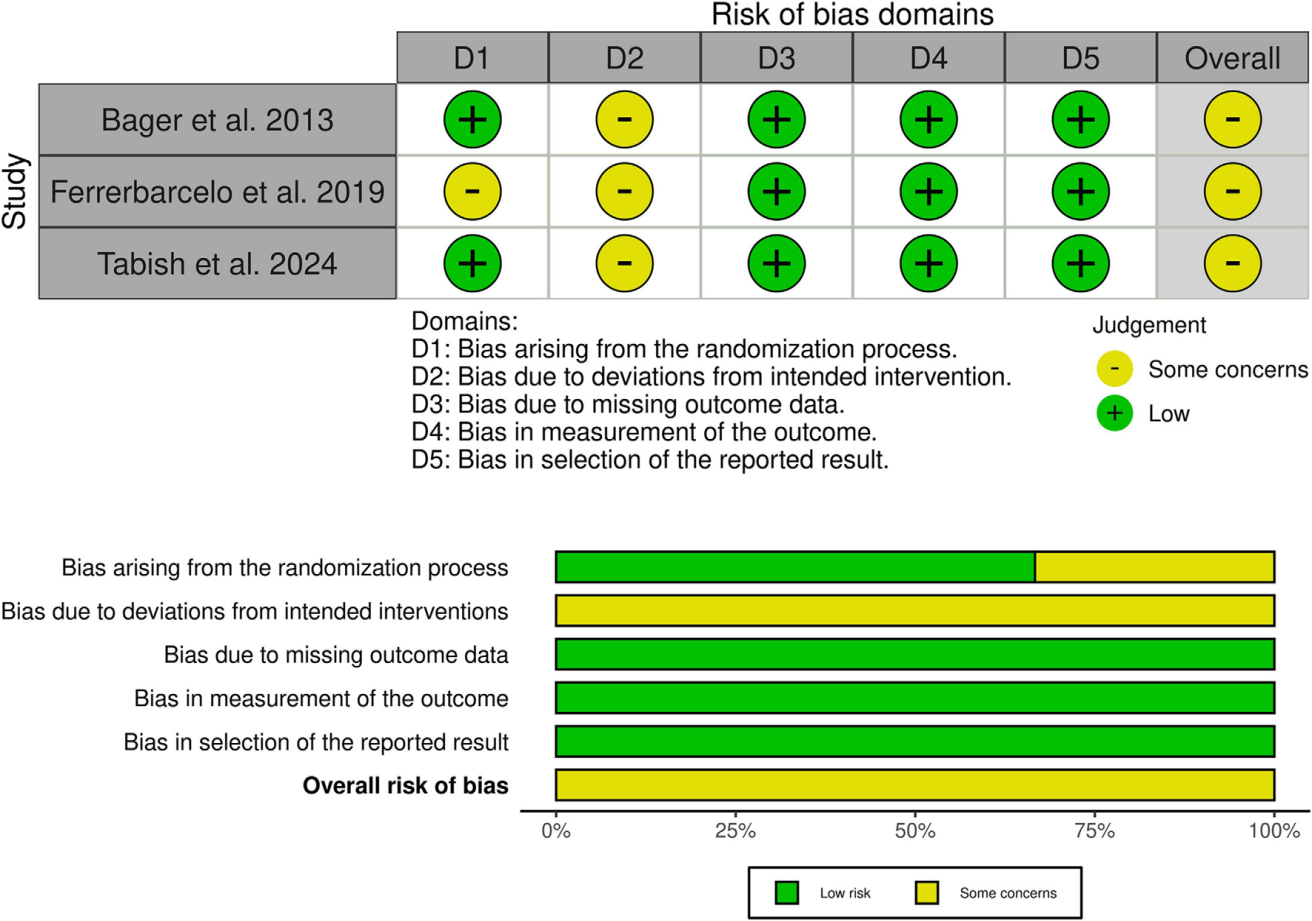
Quality assessment of risk of bias in the included trials. The upper panel presents a schematic representation of risks (low = green, unclear = yellow, and high = red) for specific types of biases of each of the studies in the review. The lower panel presents risks (low = green, unclear = yellow, and high = red) for the subtypes of biases of the combination of studies included in this review.

All the included studies showed some concerns of bias, mainly due to deviations from the intended interventions. These deviations were unavoidable because of the different routes of administration and could not be disregarded due to the open‐label design of the interventions. In particular, adherence to oral iron therapy was patient‐dependent rather than physician‐regulated. Unlike IV iron, which was administered in a supervised setting, oral iron intake relied on patient compliance, potentially leading to inconsistencies in dosing and treatment duration. Also, Ferrer‐Barcelo et al. showed some concerns about selection bias as they used alternating sequences as a randomization method, but no difference in baseline characteristics was noted (Figure 2). Certainty of evidence is outlined in a GRADE evidence profile (Table 3).

**TABLE 3:** GRADE evidence profile.

Certainty assessment						
Participants (studies) Follow‐up	Risk of bias	Inconsistency	Indirectness	Imprecision	Publication bias	Overall certainty of evidence
Complete response						
235 (3 RCTs)	Serious^a^	Not serious	Not serious	Serious^b^	None	⨁⨁◯◯ Low
Partial response						
143 (2 RCTs)	Serious^a^	Very serious^c^	Not serious	Very serious^b^	None	⨁◯◯◯ Very low
Hemoglobin change						
235 (3 RCTs)	Serious^a^	Serious^d^	Not serious	Serious^e^	None	⨁◯◯◯ Very low
Ferritin change						
137 (2 RCTs)	Serious^a^	Very serious^c^	Not serious	Extremely serious^e^	None	⨁◯◯◯ Very low
TSAT change						
138 (2 RCTs)	Serious^a^	Serious^d^	Not serious	Very serious^e^	None	⨁◯◯◯ Very low
Hypophosphatemia						
175 (2 RCTs)	Serious^a^	Not serious	Not serious	Serious^b^	Strong association	⨁⨁⨁◯ Moderate
Gastrointestinal adverse events						
235 (3 RCTs)	Serious^a^	Not serious	Not serious	Serious^b^	None	⨁⨁◯◯ Low
Rebleeding						
235 (3 RCTs)	Serious^a^	Not serious	Not serious	Very serious^b^	None	⨁◯◯◯ Very low

Abbreviations: CI, confidence interval; TSAT, transferrin saturation.

^a^ All the included trials showed some bias concerns due to deviations from the intended interventions.

^b^ A wide confidence interval that does not exclude the risk of appreciable harm‐benefit, with a low number of events.

^c^ *I* ^2^ > 75%.

^d^ *I* ^2^ > 50%.

^e^ A wide confidence interval that does not exclude the risk of appreciable harm‐benefit, with a low number of participants.

### 3.4 Primary Outcomes: Complete & Partial Response

**FIGURE 3:**
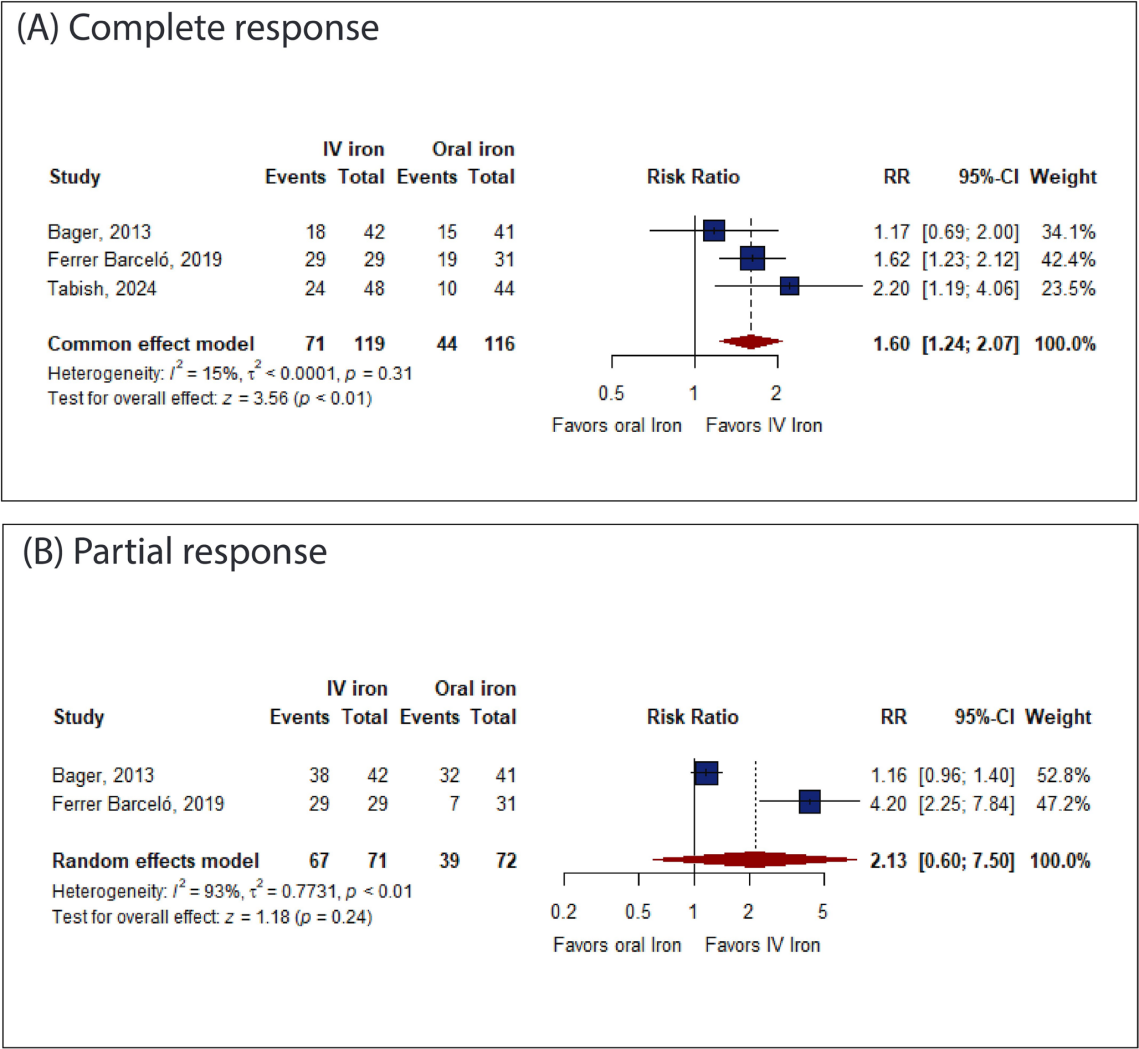
Forest plot of the primary efficacy outcomes. CI, confidence interval; RR, risk ratio.

IV iron was significantly associated with an increased complete response (RR: 1.60 with 95% CI [1.24, 2.07], *p* < 0.01) compared to oral iron (Figure 3A) with no significant difference between IV iron and oral iron in partial response (RR: 2.13 with 95% CI [0.60, 7.50], *p* = 0.24) (Figure 3B).

Pooled studies were homogeneous in complete response (*I* ^2^ = 15%, *p* = 0.31) and heterogeneous in partial response (*I* ^2^ = 93%, *p* < 0.01). Sensitivity analysis was not applicable in partial response.

### 3.5 Secondary Outcomes

#### 3.5.1 Efficacy Outcomes

**FIGURE 4:**
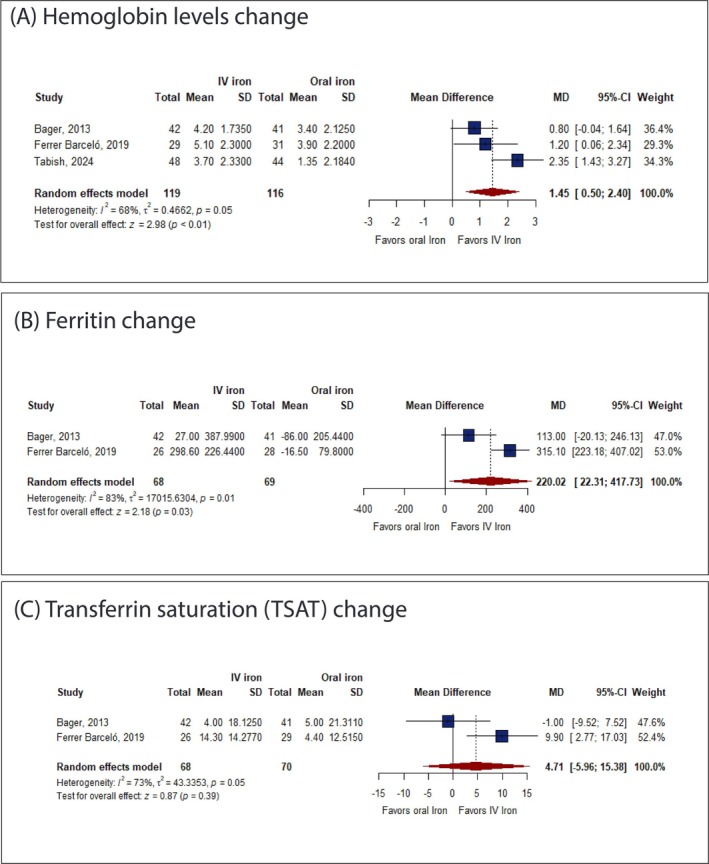
Forest plot of the secondary efficacy outcomes. CI, confidence interval; RR, risk ratio.

IV iron was significantly associated with increased Hb concentration (MD: 1.45 g/dL with 95% CI [0.50, 2.40], *p* < 0.01) (Figure 4A) and ferritin change (MD: 220.02 μg/L with 95% CI [22.31, 417.73], *p* = 0.03) (Figure 4B) compared to oral iron. However, there was no significant difference between IV and oral iron in TSAT (MD: 4.71% with 95% CI [−5.96, 15.38], *p* = 0.39) (Figure 4C).

The pooled studies were heterogeneous in Hb concentration (*I* ^2^ = 68%, *p* = 0.05), ferritin change (*I* ^2^ = 83%, *p* = 0.01), and TSAT (*I* ^2^ = 73%, *p* = 0.05). Regarding Hb concentration, heterogeneity was best resolved by excluding Tabish et al. (*I* ^2^ = 0) (see Figure S1). Regarding ferritin change and TSAT, sensitivity analysis was not applicable.

#### 3.5.2 Safety Outcomes

**FIGURE 5:**
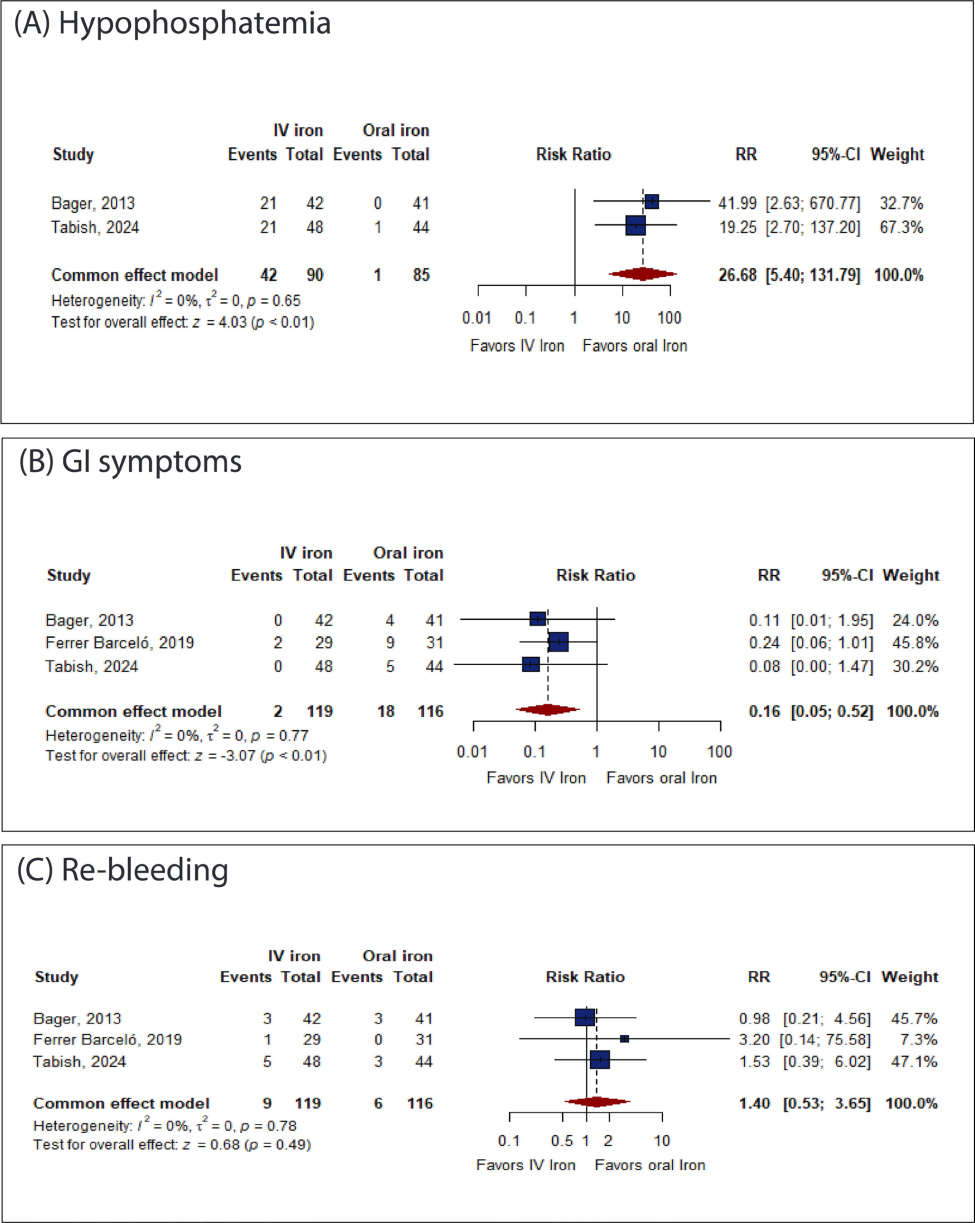
Forest plot of the secondary safety outcomes. CI, confidence interval; RR, risk ratio.

IV iron was significantly associated with an increased incidence of hypophosphatemia (≤ 2.5 mg/dL). However, most cases were mild and resolved by the end of the trial (RR: 26.68 with 95% CI [5.40, 131.79], *p* < 0.01) (Figure 5A). In contrast, IV iron was significantly associated with a decreased incidence of GI symptoms (RR: 0.16 with 95% CI [0.05, 0.52], *p* < 0.01) (Figure 5B). However, there was no significant difference between IV and oral iron in the incidence of rebleeding (RR: 1.40 with 95% CI [0.53, 3.65], *p* = 0.49) (Figure 5C).

Pooled studies were homogenous in hypophosphatemia (*I* ^2^ = 0%, *p* = 0.65), GI symptoms (*I* ^2^ = 0%, *p* = 0.77), and rebleeding (*I* ^2^ = 0%, *p* = 0.78).

### 3.6 Trial Sequential Analysis

In complete response and hemoglobin change, the cumulative Z‐curve crossed both the conventional boundary for the benefit and the trial sequential monitoring boundary, establishing sufficient and conclusive evidence and suggesting that further trials are unnecessary (see Figures S1 and S2).

## 4 Discussion

Oral iron supplementation has been the mainstay for treatment for patients with GIB, while IV iron provides a convenient and potentially safer alternative. This meta‐analysis aimed to compare the effectiveness and safety of oral versus IV iron therapy in treating patients with GIB. The pooled analysis of the three RCTs [11, 12, 13] demonstrated that IV iron significantly improved complete response, hemoglobin, and ferritin; reduced gastrointestinal adverse events; but did not improve TSAT, partial response, or rebleeding, with an increased rate of hypophosphatemia.

However, the high heterogeneity level among hemoglobin, ferritin, and TSAT was likely due to multiple factors. First, patient characteristics differed, with Bager and Dahlerup [12] and Ferrer Barceló et al. [13] incorporating patients with non‐variceal upper GIB, while Tabish et al. [11] included only variceal bleed patients who had cirrhosis, which presents a different and usually sicker population. Second, follow‐up periods differed, with 3 months, 2 months, and 7 weeks among the three studies. Third, the dosage of FCM used was 1000 mg given in the study by Bager and Dahlerup [12] and 1500–2000 mg in the other two studies [11, 13], with Tabish administering split doses [11]. Fourth, the difference in IDA status at baseline among studies may have further contributed to this heterogeneity. Finally, Tabish et al. [11] conducted an open‐label study, potentially adding biases that could have affected the outcomes.

Although no other RCTs have compared oral and IV formulations of iron in GIB, several studies have compared IV and oral formulations of iron in treating IDA. For example, the study by Onken et al. [20] demonstrated that FCM significantly increased hemoglobin by 2.9 g/dL, ferritin by 191 ng/mL, and TSAT by 16%, compared to the control group receiving oral iron. The treatment was generally well‐tolerated, with the most common adverse events being mild to moderate gastrointestinal symptoms and a notable but asymptomatic incidence of hypophosphatemia [20]. Moreover, several other studies have demonstrated IV iron's higher efficacy than oral formulations in different settings with coexistent IDA, including chronic kidney disease and cancer‐induced anemia [21, 22].

Regarding GIB specifically, several retrospective studies were conducted to assess the efficacy of IV iron in treating anemia in GIB. For instance, the retrospective study by Ballester‐Clau et al. [4] found that IV FCM effectively improved hemoglobin concentration and was well‐tolerated in patients with acute GIB, even in high‐risk groups. This aligns with our meta‐analysis results, which show the effectiveness of IV iron in rapidly increasing hemoglobin and ferritin concentration with few gastrointestinal side effects. The study by Ballester‐Clau et al. [4] also highlighted a significant reduction in the need for blood transfusions with FCM, a benefit not specifically addressed in our meta‐analysis.

On further analysis of prior data of anemic patients presenting with GIB, the results of this meta‐analysis present further advantages. Anemic patients have been shown in previous studies to have low follow‐up rates and even reduced prescription of iron repletion on discharge [23]. Not only is there scarce information about post‐discharge iron repletion and anemia follow‐up in patients with GIB, but there exists no systematic method or clear guidelines for follow‐up on iron repletion post‐discharge after a diagnosis with GIB [24]. There is often a lack of continuity between care during the acute illness and transition to the outpatient setting, often with the primary care physician handling care with no specific instructions on iron repletion or confirmation of therapy completion. This is especially the case given reports that some physicians are less likely to follow up on IDA in patients with GIB, hypothesized to possibly be due to the presence of an explanation for the anemia given the bleeding with no attention to concurrent IDA. All these factors build up to the benefit of a shorter course of repletion with IV iron formulations in the critical pool of patients such as those with GIB [23].

The clinical significance of this meta‐analysis extends beyond comparative efficacy and into reshaping how iron repletion is approached in the post‐GIB setting. Given that GIB‐related anemia often occurs in older, comorbid, or cirrhotic patients—many of whom face barriers to consistent outpatient care—the use of IV iron may serve as a strategy not just for correction of anemia, but for improving transitions of care. The ability to complete treatment during hospitalization or in a single outpatient visit reduces dependence on long‐term adherence and mitigates the risk of therapeutic inertia following discharge. Furthermore, in health systems lacking robust post‐discharge follow‐up, IV iron may function as a safeguard against the common underdiagnosis and undertreatment of persistent iron deficiency.

The safety profile of IV iron versus oral iron in treating GI bleeds was assessed based on the incidence of hypophosphatemia, GI symptoms, and rebleeding events. Patients receiving oral iron reported more frequent GI symptoms, such as nausea, vomiting, and constipation, which could impact compliance and overall treatment satisfaction, as discussed above. This is well established in multiple other studies; for instance, the meta‐analysis by Tolkien et al. [25] demonstrates the significant GI side effects of oral iron, with no difference associated with dosage. Overall, while IV iron therapy presents a higher risk of hypophosphatemia, it offers a lower incidence of GI symptoms than oral iron. Both treatments appear to have a comparable safety profile regarding the risk of rebleeding.

### 4.1 Strengths & Limitations

To the extent of our knowledge, this is the first systematic review and meta‐analysis to investigate the efficacy and safety of IV iron to replete iron after GIB. Still, our study has a few limitations: first, the small number of RCTs analyzing the question of concern, with the study by Tabish et al. [11] additionally being unblinded. Second, heterogeneity was high in multiple outcomes, likely due to the high variability in patient characteristics (including incorporating variceal and non‐variceal bleeds and difference in IDA status at baseline), duration of follow‐up, and differing dosing regimens. Third, the GRADE assessment yielded low certainty of current evidence; therefore, our findings must be interpreted cautiously. Finally, not all studies exclusively included patients with IDA (e.g., Bager and Dahlerup), limiting the ability to isolate the effect of IV vs. oral iron in pure IDA cases.

### 4.2 Implications for Future Research

Future studies should identify the heterogeneity observed in the meta‐analysis, particularly regarding variations in patient populations, dosing regimens, and baseline characteristics. Large‐scale, multicenter RCTs are needed to understand better the differential impacts of various IV iron formulations on efficacy and safety outcomes and compare dosing regimens. Additionally, future studies should explore the long‐term effects of IV iron therapy on iron stores and anemia resolution and investigate strategies to mitigate the risk of hypophosphatemia.

## 5 Conclusion

With uncertain evidence, IV iron demonstrated increased hemoglobin and ferritin concentrations and achieved complete response rates in patients with GI bleeding. However, the variability in TSAT change and partial response rate indicates a need for further studies to understand these outcomes better. The findings support the use of IV iron, especially in cases where rapid iron repletion is critical, while also considering patient‐specific factors and potential variability in response. IV iron had fewer GI side effects than oral iron while having more incidence of hypophosphatemia, with no difference being found in rebleeding events.

The results of this meta‐analysis provide essential insights into the effectiveness of IV iron in repleting iron in patients with GIB. This offers valuable directions to potentially assist in creating future guidelines for clinicians managing patients with GIB, minimizing risk, and maximizing completion of therapy with the most convenience.

## Disclosure

The authors have nothing to report.

## Consent

The authors have nothing to report.

## Conflicts of Interest

The authors declare no conflicts of interest.

## Supporting information


**Data S1.** Supporting Information.

## Data Availability

The data that support the findings of this study are available from the corresponding author upon reasonable request.
