# Supplementary material for: Intravenous Versus Oral Iron After Gastrointestinal Bleeding: A Systematic Review and Meta‐Analysis of Randomized Controlled Trials
Source: JGH Open. 2025 Jul 18;9(7):e70225. doi: 10.1002/jgh3.70225 (PMC12272139; doi:10.1002/jgh3.70225)
Supplement: Supplementary file 1 — Data S1. Supporting Information. [file JGH3-9-e70225-s001.docx]

**Supplementary Materials:**

**Title.**

**Intravenous versus Oral Iron after Gastrointestinal Bleeding: A Systematic Review and Meta-Analysis of Randomized Controlled Trials.**

**Running Title.**

Intravenous vs. Oral Iron after GIB.

**Keywords.**

Hemorrhage; transfusion; anemia; varices; variceal.

**Contents:**

**Tables.**Table S1: Search strategy.

**Figures**.

Figure M1 Forest plot of the secondary safety outcomes, RR: risk ratio, CI: confidence interval.

Figure S1: Trial sequential analysis of complete response.

Figure S2: Trial sequential analysis of hemoglobin change.

| Database | Search Terms | Search Field | Search Results |
| --- | --- | --- | --- |
| Pubmed | (“intravenous iron” OR “ferric carboxymaltose” OR “IV iron” OR ”iron derisomaltose” OR “iron supplement*” OR “iron therapy” OR “iron sucrose" OR "iron isomaltoside” OR “ferric gluconate”) AND (gastrointestinal* OR GI OR varices OR variceal OR nonvariceal) AND (bleed* OR hemorrhage OR haemorrhage) | All Fields | 550 |
| CENTRAL | (“intravenous iron” OR “ferric carboxymaltose” OR “IV iron” OR ”iron derisomaltose” OR “iron supplement*” OR “iron therapy” OR “iron sucrose" OR "iron isomaltoside” OR “ferric gluconate”) AND (gastrointestinal* OR GI OR varices OR variceal OR nonvariceal) AND (bleed* OR hemorrhage OR haemorrhage) | All Text | 87 |
| WOS | (“intravenous iron” OR “ferric carboxymaltose” OR “IV iron” OR ”iron derisomaltose” OR “iron supplement*” OR “iron therapy” OR “iron sucrose" OR "iron isomaltoside” OR “ferric gluconate”) AND (gastrointestinal* OR GI OR varices OR variceal OR nonvariceal) AND (bleed* OR hemorrhage OR haemorrhage) | All Fields | 211 |
| Scopus | (“intravenous iron” OR “ferric carboxymaltose” OR “IV iron” OR ”iron derisomaltose” OR “iron supplement*” OR “iron therapy” OR “iron sucrose" OR "iron isomaltoside” OR “ferric gluconate”) AND (gastrointestinal* OR GI OR varices OR variceal OR nonvariceal) AND (bleed* OR hemorrhage OR haemorrhage) | Title, Abstract, Keywords | 783 |
| EMBASE | #1 AND #2 AND #3   #1. 'intravenous iron' OR (intravenous AND ('iron'/exp OR iron)) OR (iron AND sucrose) OR 'iron therapy' OR (iron AND isomaltoside) OR (iron AND supplement*) OR (iron AND derisomaltose) OR (ferric AND carboxymaltose) OR (iv AND iron) OR (ferric AND gluconate)  #2 gastrointestinal OR gi OR varices OR nonvariceal OR variceal  #3. bleed OR hemorrhage OR haemorrhage | All Fields | 1,824 |

Table S1: Search Strategy.


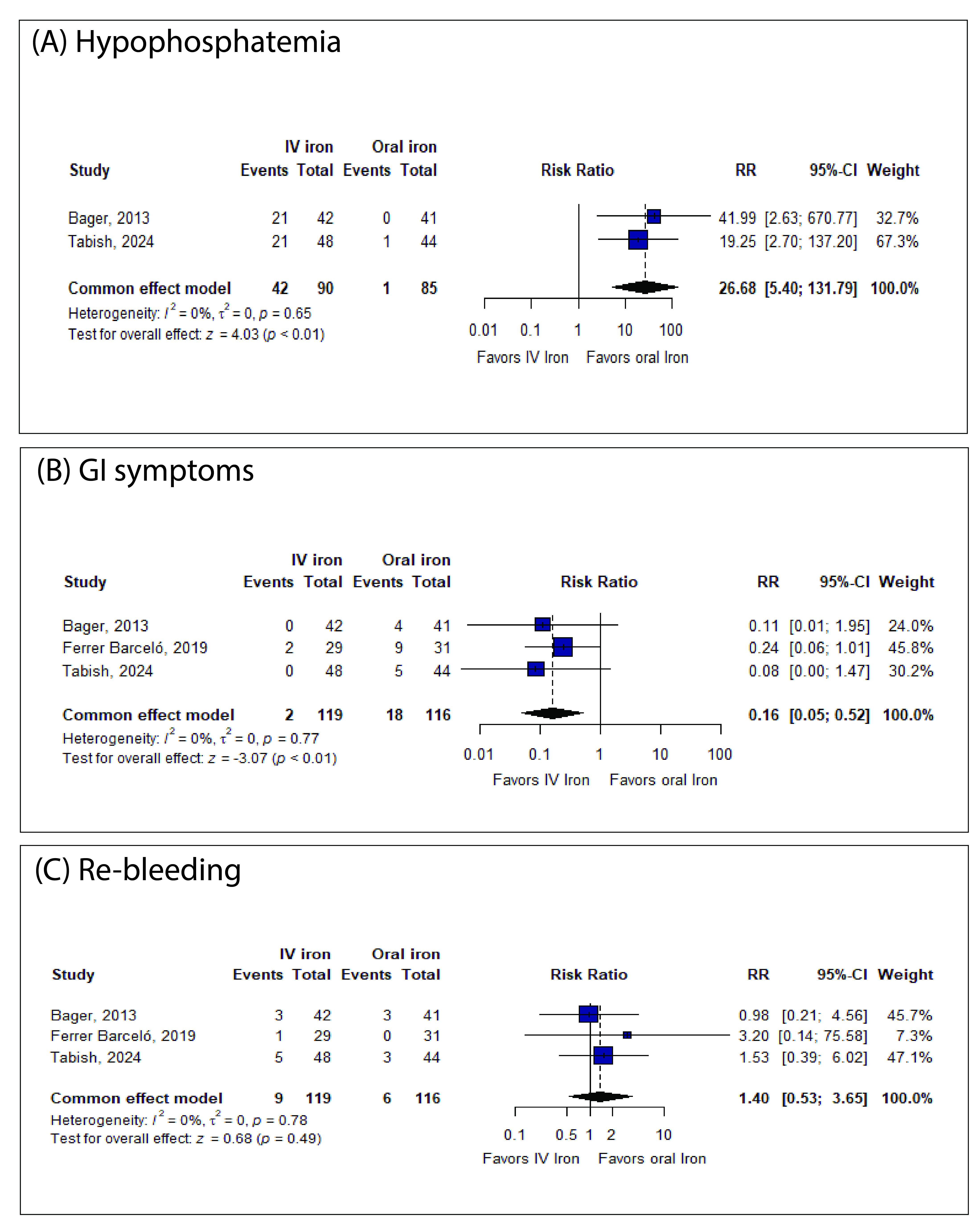


*Figure M1 Forest plot of the secondary safety outcomes, RR: risk ratio, CI: confidence interval.*


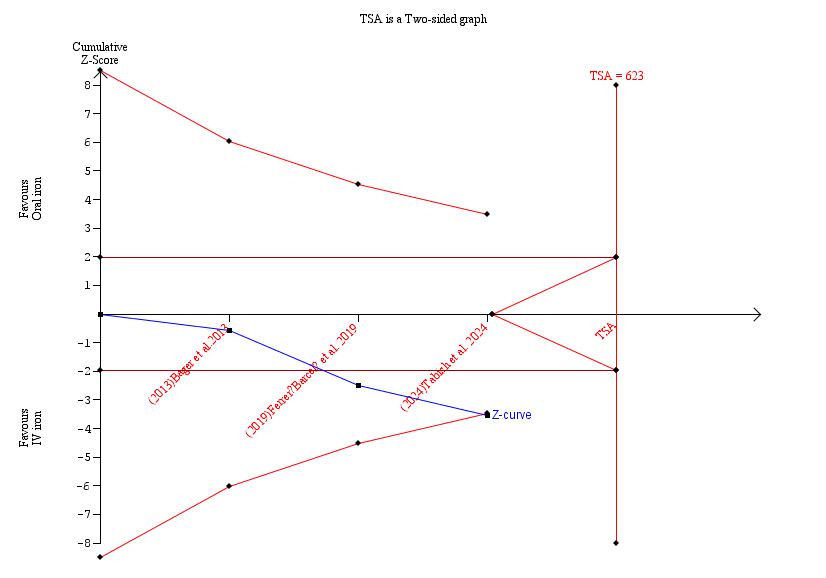


Figure S1: Trial sequential analysis of complete response.


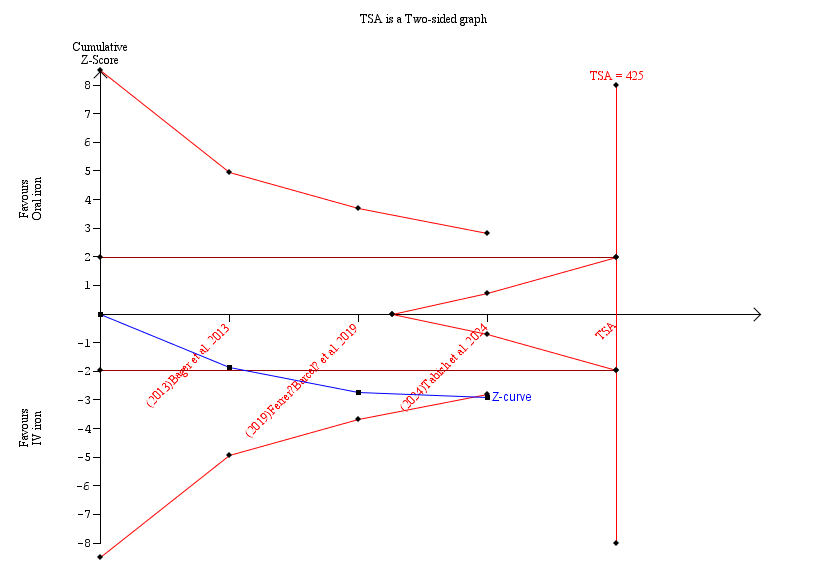


Figure S2: Trial sequential analysis of hemoglobin change.
